# Sub-Saharan African migrant youths’ help-seeking barriers and facilitators for mental health and substance use problems: a qualitative study

**DOI:** 10.1186/s12888-016-0984-5

**Published:** 2016-08-02

**Authors:** Terence V. McCann, Janette Mugavin, Andre Renzaho, Dan I. Lubman

**Affiliations:** 1 Discipline of Nursing, Centre for Chronic Disease, College of Health and Biomedicine, Victoria University, PO Box 1428, Melbourne, 8001 VIC Australia; 2 Turning Point and Eastern Health, Melbourne, Australia; 3 Centre for Alcohol Policy Research, La Trobe University, Melbourne, Australia; 4 Western Sydney University, Sydney, Australia; 5 Monash University, Melbourne, Australia

**Keywords:** Sub-Saharan African migrants, Barriers, Facilitators, Focus groups, Help-seeking, Individual interviews, Mental health problems, Qualitative research, Refugees, Substance use problems

## Abstract

**Background:**

Many young migrants and their parents are reluctant to seek help for mental health and substance use problems. Help-seeking delays can result in longer duration of untreated problems and poorer outcomes. In this study, we aimed to identify the help-seeking barriers and facilitators for anxiety, depression and alcohol and drug use problems in young people from recently established sub-Saharan African migrant communities.

**Methods:**

A qualitative study, incorporating individual, in-depth interviews and focus group discussions, was undertaken in Melbourne, Australia. Twenty-eight young sub-Saharan African migrants participated in the individual interviews, and 41 sub-Saharan African-born parents and key community leaders participated in 4 focus groups. All participants were aged 16 years or over. A thematic analysis of the data was undertaken.

**Results:**

Themes and related sub-themes were abstracted from the data, reflecting the young people’s, parents’ and key community leaders’ beliefs about barriers and facilitators to help-seeking for mental health and substance use problems. Four help-seeking barriers were identified: stigma of mental illness, lack of mental health literacy in parents and young people, lack of cultural competency of formal help sources, and financial costs deterring access. Five help-seeking facilitators were abstracted: being open with friends and family, strong community support systems, trustworthiness and confidentiality of help-sources, perceived expertise of formal help-sources, increasing young people’s and parents’ mental health literacy.

**Conclusion:**

Programs that identify and build on help-seeking facilitators while addressing help-seeking barriers are needed to address mental health issues among young sub-Saharan African migrants. Strategies to address help-seeking barriers should consider counteracting stigma and increasing mental health literacy in sub-Saharan African communities, increasing health providers’ cultural competency and perceived trustworthiness, and addressing financial barriers to accessing services.

## Background

More than a quarter (27.7 %) of Australian residents were born overseas [1]. For many new migrants, adjusting to life in a new country presents a range of challenges; for example, learning a new language, adjusting to different cultural and societal norms, coping with financial difficulties, and having fewer family and community connections. Migration presents unique challenges for young people and parents. Different rates of acculturation (cultural and psychological changes that occur when two cultures come into contact with each other) [2, 3] between young migrants and older family members, with young people frequently adjusting at a faster rate [4, 5], may place additional challenges on families, including responding to mental health and substance use problems. In Australia, youth mental health is a significant concern, with around 1-in-4 young people (aged 16 to 24 years) experiencing at least one mental disorder (most frequently anxiety, affective and/or substance use disorders) in the preceding 12 months [6]. However, less than 1-in-4 young people with mental health problems seek professional help [7], which is problematic as help-seeking delays can have a marked adverse effect on educational, vocational and social outcomes, and may have detrimental long-term consequences [8]. Young migrants are vulnerable to the risk factors affecting youths in general, but also experience additional pressures that place them at risk of adversity, such as problems of acculturation and racism, difficulties that are also associated with an increase in alcohol and drug use problems ([9–11] p. 20). Moreover, many young migrants, especially refugees [12], who experience mental health and alcohol and drug use problems are reluctant to seek help [13]. A UK study of help-seeking in African-Caribbean women found that even though these women had high levels of psychosocial risks, they were relatively invisible in seeking and receiving help for perinatal depression compared to other women in this situation [14].

Help-seeking delays can result in longer duration of untreated illness and poorer outcomes [15]. While migrants are not a homogenous group concerning help-seeking for mental health problems, help-seeking delays are common in these communities [16, 17]. This was evidenced in a Netherlands study where undocumented migrants were more likely to approach informal help sources, such as friends and religious institutions, than formal help sources [16], and in a review of literature where the legal frame of the host country and language barriers deterred help-seeking [17]. Help-seeking behaviours among migrant groups are affected by numerous influences, although for mental health and alcohol and drug problems, poor mental health literacy (knowledge and beliefs that assist in recognising, managing and preventing mental health issues) is particularly important [18]. Different ways of conceptualising mental health and alcohol and drug problems, accompanied by limited knowledge of services and treatment options, are also likely to affect help-seeking adversely [19, 20]. Indeed, a qualitative study of key informant workers’ from health, welfare and drug treatment services perceptions about ethnic community members’ access to alcohol and drug treatment in eight migrant communities in the Australian state of Victoria [21] identified several help-seeking barriers: services' insufficient knowledge of the perceptions, expectations and needs of their clients as well as of the diversity of people accessing their services; language barriers; lack of advocacy by community leaders; community pressures to be discreet about drug use; a desire to be self-sufficient in dealing with alcohol and drug problems; and lack of family inclusion in alcohol and drug programs. Similarly, low priority placed on addressing mental health issues by new migrants, their poor mental health literacy, lack of knowledge and distrust of services, and social and cultural barriers have detrimental effects on help-seeking to mental health services [13].

Self-stigma of mental illness, internalisation of the public stigmatisation of mental illness and consequential diminished self-esteem and self-efficacy [22], also deters help-seeking in migrant communities. Stigma associated with reporting mental health and alcohol and drug problems, and the desire to avoid bringing shame on the family and/or community provides a powerful incentive among young migrants not to disclose problems [13, 23–25]. In addition, young migrants are often reluctant to approach mental health services within their own community because of shame and concern about breach of confidentiality [13, 21]. As a consequence, many young migrants are caught in a paradoxical situation as they are often unwilling to access services within their own community and are reluctant to approach mainstream mental health services.

In addition to difficulties associated with poor mental health literacy and stigma, young migrants experience several other risk factors, including the stress of forced or voluntary migration and the process of resettlement. There may also be a history of torture and other trauma, low income, lack of meaningful work, difficulties at school, and a desire to gain acceptance through participation in the drinking patterns of young people who were born in the country [26, 27]. Furthermore, their family may be absent, or there may be family conflict as a result of acculturation difficulties [9]. While adults migrants tend to continue to maintain traditional values and norms from their culture of origin, their children often embrace the cultural attitudes and behaviours of the host country, meaning these young people are at risk of alienation from both cultures [19]. Differential family acculturation, role reversal and loss of parental control over adolescents by parents pose a threat to the well-being of young migrants and family members and, thus, increase a young person’s susceptibility to mental health problems [5, 24, 28, 29].

While help-seeking for mental health and substance use problems has been relatively well documented in older adults, and to a lesser extent in young people in general [30], considerably less is known about intergenerational issues associated with the help-seeking experience of young migrants from sub-Saharan African countries where help-seeking delays occur. Hence, it is important to understand the help-seeking barriers and facilitators for mental health and substance use problems that young people from these countries encounter as delays can result in longer duration of untreated problems and poorer outcomes. Unless such intergenerational differences are well understood, reducing migration-related inequalities in accessing mental health and alcohol and drug services will remain a challenge. In this study, we aimed to identify help-seeking barriers and facilitators for anxiety, depression and substance use problems in young people from recently established sub-Saharan African migrant communities. The term migrant is used in this study to encompass permanent migrants, those arriving in Australia under the Migration Program for skilled and family entrants and the Refugee and Humanitarian Program [31].

## Method

An inductive qualitative approach [32] was adopted to explore issues associated with help-seeking in sub-Saharan African migrant communities. This paradigm affords a rich and in-depth insight into this under-researched issue. Specifically, a triangulated approach was used, incorporating data (young people, parents and community leaders) and methodological (qualitative individual interviews and focus groups) triangulation [32, 33]. Triangulation enhanced data richness or completeness by enabling the researchers to explore a broad range of perspectives and compare and contrast perspectives, about the phenomenon (barriers and facilitators influencing help-seeking). Moreover, triangulation provided a more comprehensive understanding of the phenomenon than could be achieved by using a single data source or data collection method [34].

Audio-recorded individual interviews were carried out with the young people and focus group discussions were undertaken with parents and key community leaders.

### Participants and procedure

Purposive sampling [35] informed participant recruitment. Inclusion criteria for the young people were: (i) sub-Saharan African migrants residing in Melbourne, (ii) aged between 16 and 25 years, and (iii) first-hand experience and/or awareness of mental health and/or substance use problems among young people within their community (i.e., through personal, family or peer experience). Inclusion criteria for the adults were: sub-Saharan African born migrant parents and community leaders. In light of data triangulation, where we wished to obtain a wide range of experiences about these issues, the focus groups contained parents of young people who participated in the individual interviews as well as parents of young people who did not. As the majority of the focus group participants were parents, the term ‘parents’ is used to reference data from these sources.

The researchers worked with the African Review Panel, a community-owned steering committee that oversees a number of research projects among African migrants. The Panel is made of African workers and community leaders. The role of the panel was to facilitate access to African communities. Participants were located through community structures, including churches, African youth clubs, community health workers, and African community organisation networks.

Semi-structured interview and focus group discussion guides were developed from a review of literature, discussion among the researchers, and consultation with the African Review Panel. Individual interviews were conducted in English as the young participants were proficient in conversational English, and took place in the participant’s home or a mutually convenient location. Two bilingual African-born research assistants, who recruited participants through community networks and two youth specific mental health and alcohol and drug services, conducted the interviews. Each interview lasted between 20 and 90 min. Open-ended were used to explore seven overarching topics: young people’s experience of being a new migrant in Australia; young people’s understanding of mental health and alcohol and drug use problems in their communities; help-seeking options and awareness of services; what assisted or encouraged a young person to seek help; and what factors impeded a young person seeking help. To minimise the likelihood of interviewer bias, the research assistants underwent interviewer training beforehand, were requested to adhere flexibly to the interview guide, and, together with the researchers, participated in a review process of the first set of audio-recorded interviews.

Three bilingual research assistants recruited focus groups participants. Focus groups were held in community centres and a public park. Discussions were facilitated by senior investigators, with the support of one of the bilingual research assistants who recruited participants. In addition to audio recordings, field notes were taken. Three of the four focus groups were conducted in English; one was conducted in Kirundi and, subsequently, translated into English. Discussion themes included: experiences of being a migrant parent in Australia, including mental health and alcohol and drug use problems among young people in their community; sources of help and help-seeking practices; and preferred mode of receiving health-related information. Focus group discussions lasted between 90 and 120 min. At the end of each main section of the discussion, the researcher summarised the content to ensure the participants’ perspectives were obtained and interpreted correctly, a verification process that enhanced the credibility of the findings [36].

Each study participant received a $25 supermarket voucher as reimbursement for their time, inconvenience and any associated travel costs.

### Data analyses

Mindful of the overarching framework in which the questions were asked (help-seeking barriers and facilitators), we used Braun and Clarke’s six-step approach to analyse and develop themes inductively from the data [37]. Initially, interview and focus group data were analysed separately and subsequently combined. (i) Familiarisation with the data. Transcripts (audio recordings and field notes) were read and re-read to obtain a broad understanding of participants’ beliefs about help seeking, and initial ideas were noted. (ii) Generating initial codes. Transcripts were examined closely and initial codes inserted. (iii) Searching for themes. Codes were grouped into potential themes. (iv) Reviewing themes. Themes were reviewed and a thematic ‘map’ of the analysis was generated. (v) Defining and naming themes. Themes were refined and grouped into themes and sub-themes. Saturation of themes with ‘thick’ description of the data was researched when no new data emerged to support each theme [38] a key criterion of the rigour of qualitative methods in determining sample size [39, 40]. (vi) Producing the report. Selection of illustrative exemplars for each theme and producing a scholarly report occurred. Finally, the Hill et al. [41] criteria were adopted to determine theme representativeness to the study participants: ‘general’ — applies to all or all but one of cases; ‘typical’ — relates to half or more cases up to the cut-off for general; ‘variant’ — applies to more than two but less than half the cases; (for samples greater than 15) ‘rare’ — applies to 2-3 cases; and those that apply to only one case are not reported.

Preliminary thematic analysis was undertaken by JM. This was followed by an independent review of the process by TMcC, DL and AR, an activity that improved the rigour of the study [35]. Differences in coding and theme identification were overcome through discussion. A semantic level of analysis was undertaken, progressing from initial description and summary, in the results section, to interpretation, in the discussion section [37].

## Results

Twenty-eight young people took part in the individual interviews. Approximately two-thirds were male (*n* = 18, 64.3 %) and one third were female (*n* = 10, 35.7 %), and their mean age was 20 years (SD 3.7). Twenty-five (89 %) had lived in Australia for 6 years or less, while 3 (11 %) had resided in the country for 11–19 years. Forty-one sub-Saharan African-born parents and key community leaders took part in 4 focus groups (FGs) (FG1, *n* = 10; FG2, *n* = 9; FG3, *n* = 11; FG4, *n* = 11). Almost 60 % were male (*n* = 24, 58.5 %). Nine (22 %) participants had lived in Australia for 5 years or less, while 32 (78 %) had resided in the country for 6–10 years.

### Help-seeking barriers

Four help-seeking barrier themes were abstracted from the data, highlighting deterrents to accessing informal and formal support for mental health and alcohol and drug use problems: stigma of mental illness, lack of mental health literacy in parents and young people, perceived lack of cultural competency of formal help sources, and financial costs deterring access.

#### Stigma of mental illness

In this general theme, stigma associated with disclosing mental health and alcohol and drug problems was reported frequently. Stigma was influenced by a desire to avoid bringing shame on their family and community and provided a powerful incentive not to disclose these problems. Fears associated with disclosure meant that the young people and parents were often reluctant to seek help within their specific sub-Saharan African migrant community. Help-seeking from informal or formal sources was perceived as sign of personal weakness or failure. As such, feeling ashamed of one’s own mental health or alcohol and drug problem may deter young people from seeking help:

The shame. The shame in this community again; being labelled a drunkard. It's like I failed myself; I had to go and seek extra help. So it's the shame; the disappointment would stop me; the embarrassment (Interviewee 20).

Stigma posed a particular problem for parents, as informal community networks were their preferred way of addressing other health issues. Stigma was also identified as an obstacle to seeking help from professional services.

I don’t know about other cultures but my understanding in my culture is, well as an individual, I might recognise these [mental health specialists] are qualified professionals in their field, but I grew up knowing them as ‘shrinks.’ So I wouldn’t want to be seen outside the door of one; that’s my belief (Focus Group 1 participant).

#### Lack of mental health literacy in parents and young people

In this general theme, both sets of participants commented about poor mental health literacy regarding early problem recognition and the availability of professional support and treatment, particularly among parents. Among young people, dismissing early signs of alcohol-related problems, and only seeking help when problems became serious, were common.

… the reason why [young] people who are mentally ill - or using drugs - the reason why they don't go to specialists I think is because they think they’re not sick. They don’t think they are sick themselves so there’s no point of going to the specialists (Interviewee 5).

According to the young people, parents’ perceived low level of mental health literacy meant that young people may not approach them to seek help with mental health and alcohol and drug use problems.

… honestly speaking, back in … [country of origin], it’s not like you can pick a person who is schizophrenic over there, that one has schizophrenia, that one has depression, that one is manic. You cannot pick that. So it’s not a topic that is discussed every day, it’s not. It could be that people are not even aware that this is a mental illness happening right here, even if they see the symptoms, they don’t even know. They think, “well, you know what, this child is just being stubborn, they are just doing this, and they are not listening.” That is what they think (Focus Group 3, participant).

#### Perceived lack of cultural competency of formal help sources

In this typical theme — emphasised by parents in particular — a shortage of same-culture health professionals was perceived as a help-seeking barrier. Participants commented that, unless the health professional was born in Africa, or had extensive understanding of African cultures, there was limited value in seeking formal help as the cultural context of the mental health, alcohol and drug use problem was unlikely to be taken into consideration. Treating the issue within a culturally-sensitive framework was also viewed as an essential part of the healing process. Across the focus groups, few parents were aware of mental health or alcohol and drug services that employed an African health professional. However, a few were confident they could find a same-culture health professional, if required.

I would so much appreciate access to information of culturally-sensitive psychologists or [health] professionals. Sometimes we think twice about going to see a professional because by the time you get through the cultural understanding it will be seven months down the line and my child will have done whatever they wanted to do (Focus Group 1, participant).

#### Financial costs deterring access

In this typical theme, the financial cost of seeking formal help was viewed as a barrier by parents and young people, with the latter suggesting the perceived costs of seeing a specialist would deter parents from seeking formal help for their son or daughter. Within a sub-Saharan African context, the ability to access specialists was regarded as a sign of financial wealth, as specialist services were often only accessible through private health systems, which were unaffordable to most people.

… the costs, like health costs, medical expenses. If they can be reduced then more parents would be able to afford. Because some parents may not be able to take their children for much help because of the costs …. if it’s too expensive, they’ll not definitely go there; can’t afford it (Interviewee 10).

Even though financial costs were a deterrent to accessing specialist services, in some instances the young people were able to circumvent this barrier.

Actually, in our university, … we can get a free psychologist. Because I was not able to afford the cost of a psychologist, so that’s why I decided to go to the university to ask if they have the psychologist. … I went there [to see the university psychologist] and it was free; so I benefitted from it (Interviewee 8).

In addition to financial costs, there was an incorrect perception by some participants that Australian mental health and alcohol and drug specialist services were not accessible to individuals holding a Refugee Visa, in contrast to those holding other Permanent Resident Visas or Citizenship.

### Access facilitators

Five help-seeking facilitator themes were abstracted from the data, reflecting influences that enabled access to informal and formal support for mental health and alcohol and drug use problems: being open with friends and family, strong community support systems, trustworthiness and confidentiality of help-sources, perceived expertise of formal help-sources, increasing young people’s and parents’ mental health literacy.

#### Being open with friends and family

In this typical theme, participants felt established relationships, familiarity of close friends and the bond between and within families facilitated help-seeking through informal sources. As a consequence, they felt comfortable about sharing personal information with friends or family, because they were accessible, ‘they are always around,’ and were perceived as being approachable and supportive with the young people’s mental health and alcohol and drug use problems. In addition, the personal knowledge and insights of friends and family meant they were well placed to recognise problems and offer support, encouragement and advice. For young people, encouragement and emotional support provided by friends and family also facilitated access to formal help sources.

Actually, if it’s a friend, a friend means a lot. If you’re best friends, you talk a lot and you know a lot about each other. So if I go to my friend, I know they will understand how I feel and they will understand what is best for me (Interviewee 8).

#### Strong community support systems

In this typical theme, well-established support systems and networks within sub-Saharan African communities were viewed by young people and parents as a protective factor against young people developing mental health and alcohol and drug problems as well as an avenue through which emotional and practical help and advice could be sought and obtained from informal and formal sources. Strong cultural affiliations enabled parents and young people to capitalise on existing resources within their own community and were a means of providing access to professional support. The positive impact of cultural support systems and structures was thought to increase over time as their communities became more established and members were better positioned to balance the cultural values and practices of their country of origin with those in Australia.

As a community, we have tried, since the time we arrived here, to be available for each other, to be a support group for each other, because no one understands us better than our own (Focus Group 3, participant).

#### Trustworthiness and confidentiality of help-sources

In this typical theme, and within the context of mental health and alcohol and drug use problems being highly stigmatised in the general community, the trustworthiness of help sources, and assurance that information disclosed would remain confidential, were critical considerations in the help-seeking process. For the most part, trustworthiness was discussed in reference to seeking help from informal sources.

At first, you look at people you know … how close are you to them, how credible are they? If they have similar issues, okay. You also hope to be discrete ‘in the shadow’ (seeking help without others knowing). So it’s not like it’s a ‘free-for-all’ and you just put it on the ‘notice board’ (Focus Group 1, participant).

Confidentiality was reported commonly in reference to professional codes of conduct governing health professionals’ practice, and for this reason, young people felt they would prefer to seek help from a formal help source as opposed to a family member, friend or community member, who may breach their trust. For some young people, school teachers, priests and religious pastors were a preferred help source because confidential counselling was perceived to be part of their professional and spiritual roles.

Like, personally, I would go to a staff member [teacher] first before a friend but most people would go to a friend first. In my community I guess the teenagers trust their friends more so than anybody else and considering that most people my age spend on average, what, 30 h a week with their teachers it’d probably be a staff member second or first (Interviewee 11).

#### Perceived expertise of formal help-sources

In this typical theme, the perceived expertise of health professionals in dealing with mental health and alcohol and drug use problems, or general health problems increased some young people’s confidence in seeking help from formal services. In essence, the combined experience, knowledge and skills of health specialists and General Practitioners (GPs) were valued and reported as reasons for seeking help from formal sources. Furthermore, the ability of specialists to conduct an assessment and identify problems provided young people with a sense of assurance. This was the case for young people seeking help about alcohol and drug problems, and to a lesser extent, mental health problems.

They [specialists] have the knowledge and they know what they’re doing when they are dealing with young people. They have experience basically in that field (Interviewee 22).

#### Increasing young people’s and parents’ mental health literacy

In this general theme, while it was acknowledged that informal sources may not have specialist training or formal experience about mental health and alcohol and drug use problems, it was important, especially for parents, that these sources had some understanding of the issues. Parents and young people suggested that providing parents with education and training about mental health and alcohol and drugs would increase their confidence in managing and supporting their children. In turn, this information may facilitate help-seeking.

Even if we empowered them and gave them knowledge, if the kids are not part of education, it will go in one ear and get out the other ear; they [kids] will still be rebellious. Whereas if they are part of the training, then at least they can say we have the same journey. They can start making sense …. education in any form needs to take into account intergenerational issues; both parents and children (Focus Group 2, participant).

However, because of the high level of stigma associated with these issues, and in order to make education and training accessible and acceptable to new sub-Saharan African migrants, there was consensus that it would be more feasible to provide it within the context of maintaining general well-being rather than a sole focus on mental health and alcohol and drug related issues.

It can be a general well-being forum, which would have topics like alcohol… (Focus Group 1, participant).

Parents in particular recommended that education and training should be provided through group-based discussion forums. They also commented that as some migrant communities, such as Vietnamese, were longer established than their own, it would be beneficial to harness their experience, knowledge and skills in dealing with mental health and alcohol and drugs use problems in their young people.

You can always learn from somebody. I think it’s a good idea [to learn from other cultures] because you might only speak with other Africans and, maybe, they only have limited knowledge about different issues. But if you listen to other people, they might have different experiences and ideas, so together you learn more (Focus Group 1, participant).

## Discussion

In this exploratory study, we identified help-seeking barriers and facilitators for mental health and substance use problems in young people from recently established sub-Saharan African migrant communities. We found that self-stigma of mental illness acts as an access barrier to informal and formal help sources, which is consistent with the current literature [22, 42]. A recent systematic review of the influence of stigma on help-seeking [43] is consistent with our findings, especially among ethnic minority communities, young people and males. Our finding that migrants are often reluctant to seek help from informal and formal sources because of the stigma associated with mental health and substance use problems, and the wish to avoid bringing shame on their families and communities, is consistent with other studies [13, 23–25, 44]. In addition, a cultural mistrust of Western mental health services [45], especially by refugees who have experienced torture and other trauma [26, 27], deters help-seeking from formal sources. Stigma is also more common in individuals and communities that are disempowered and experience multiple disadvantages [46], such as some migrant communities [45]. Ways to counteract self-stigma and strengthen self-esteem and self-efficacy are to take part in activities that enhance an individual’s sense of self-determination and goal directedness [47].

Poor mental health literacy was also identified as a help-seeking barrier in the present study. This finding is somewhat understandable as a combination of self-stigma of mental illness, language and cultural barriers [21] mean that some migrant groups have poor mental health literacy, including limited knowledge of services and treatments [45, 48]. Poor mental health literacy is likely to have a detrimental effect on help-seeking [13, 19, 20], and was reported in two studies involving African participants. A US study of African-Americans’ mental health literacy and service delivery needs found that maintaining discriminatory cultural and traditional beliefs about mental illness also acted as a help-seeking barrier [42]. Similarly, a survey of the general public in Cape Town reported that poor mental health literacy and stigma of mental illness deterred help-seeking from formal help sources [49]. Improving mental health literacy is one approach to encourage early help-seeking for mental disorders [50], and is supported by our findings, with increasing young people’s and parents’ mental health literacy identified as important for facilitating help-seeking.

Perceived lack of cultural competence (‘a set of skills or processes that enable mental health professionals to provide services that are culturally appropriate for the diverse populations that they serve’ ([51], p.4)) of formal help sources was also identified as a help-seeking barrier in the present study. While migrants’ cultural background can influence their help-seeking practices, lack of cultural competence by mental health clinicians and services can have a detrimental effect on migrants’ help-seeking [51] and contribute to underuse of services [12]. Migrants’ perceptions of the need for help for mental health problems are dependent on their social embeddedness and how mental health professionals perceive them and their needs. Hence, initiatives to promote help-seeking in this cohort need to focus on a collaborative approach between health providers and migrants [14]. Moreover, in Australia, it has been recommended that mental health services need to reassess their narrow focus on ‘white middle-class Australia’ and place greater emphasis on increasing public awareness campaigns, services and research that targets ethnic communities [52].

Financial costs associated with accessing and receiving interventions were also identified as a help-seeking barrier in the current study. The cost of mental health services is a well-recognised access barrier in people with mental health problems [53, 54], and has also been reported in other studies of barriers to health care in migrants from sub-Saharan African countries [48]. Financial barriers include the cost of accessing GP services, which, in turn, creates an initial obstacle to accessing psychiatrists and the rest of the mental health system [54].

Regarding help-seeking facilitators, being open with friends and family and harnessing strong community support systems are important informal help-seeking enablers. These, in turn, can give rise to help-seeking from formal sources. Being open, openly disclosing mental health and substance use problems, is premised on the existence of established trusting relationships [55]. The importance of being open is it enables young people to cope and obtain support from family and friends and the community in general, as has been highlighted in a study of young people with depression accessing mental health services [55]. As a coping strategy, being open enables young migrants to build a social buffer against the stigma of mental health and substance use problems and helps reduce their isolation. However in the present study, some young people had to be circumspect about their openness to community members, a situation also reported by McCann et al. in their study of young people with depression [55]. Their cautiousness was further compounded by the issue of trauma in this population, and its impact on their ability to trust people.

Perceived trustworthiness, confidentiality and expertise of help-sources were important help-seeking facilitators in the present study. Concerns about confidentiality are linked with access to services. In an Australian context, young migrants may be reluctant to approach service providers within their own community because of shame and concerns about breach of confidentiality [13, 21]. The importance of a trusting and confidential relationship with school counsellors as access mediators was highlighted in the findings of an Australian study about young people with depression accessing services [30]. Concern about confidentiality breaches was also reported in a US context, where African American youths’ concerns were linked with lower levels of mental health service engagement [56]. Youth-friendliness is another important help-seeking enabler and for maintaining contact with mental health clinicians. Characteristics of youth-friendliness include clinicians developing open and friendly engagement, responding promptly to their engagement problems, and ensuring continuity of appointments with the young people [57].

### Limitations

There are several limitations to this study. As this is an exploratory qualitative study the findings are context bound to the participants and context in which the study was conducted [58]. However, even though generalisability is not a primary consideration in qualitative research [59], the themes can be verified [60] and are applicable to young sub-Saharan African migrants, parents and key community leaders in other contexts. There was an imbalance between the number of young male and female interview participants. Furthermore, the recruitment process may have resulted in an atypical sample of engaged youth and parents with a different experience to those who were less engaged with their communities.

## Conclusions

Our findings have implications for recently established migrant communities, health care providers, policy makers and governments. Measures need to be taken to promote help-seeking for these problems, including addressing stigma and improving mental health literacy in sub-Saharan African communities; increasing the cultural competency and perceived trustworthiness of health service providers, especially mental health and alcohol and drug services; and tackling financial barriers to accessing services. Furthermore, mental health and alcohol and drug related policies need to place greater emphasis on meeting the needs of sub-Saharan African (and other newly established) migrant communities; in particular, placing emphasis on the provision of culturally competent care and providing a framework to ensure more equitable access to services by migrants. One way of helping to provide more accessible and culturally competent care in this context is to encourage and support members of sub-Saharan African (and other) migrant communities to undertake education and training to work in these fields of practice.

## Abbreviations

FGs, focus groups; GPs, General Practitioners
